# A novel downstream process for highly pure 1,3‐propanediol from an efficient fed‐batch fermentation of raw glycerol by *Clostridium pasteurianum*

**DOI:** 10.1002/elsc.202100012

**Published:** 2021-05-07

**Authors:** Chijian Zhang, Shubhang Sharma, Wei Wang, An‐Ping Zeng

**Affiliations:** ^1^ Institute of Bioprocess and Biosystems Engineering Hamburg University of Technology Hamburg Germany; ^2^ Hua An Tang Biotech Group Co., Ltd Guangzhou P. R. China

**Keywords:** 1,3‐propanediol, esters, hydrolysis, salts removal, vacuum distillation

## Abstract

An efficient downstream process without prior desalination was developed for recovering 1,3‐propanediol (1,3‐PDO) with high purity and yield from broth of a highly productive fed‐batch fermentation of raw glycerol by *Clostridium pasteurianum*. After removal of biomass and proteins by ultrafiltration, and concentration by water evaporation, 1,3‐PDO was directly recovered from the broth by vacuum distillation with continuous addition and regeneration of glycerol as a supporting agent. Inorganic salts in the fermentation broth were crystallized but well suspended by a continuous flow of glycerol during the distillation process, which prevented salt precipitation and decline of heat transfer. On the other hand, ammonium salt of organic acids were liberated as ammonia gas and free organic acids under vacuum heating. The latter ones formed four types of 1,3‐PDO esters of acetic acid and butyric acid, which resulted in yield losses and low purity of 1,3‐PDO (< 80%). In order to improve the efficiency of final 1,3‐PDO rectification, we examined alkaline hydrolysis to eliminate the ester impurities. By the use of 20% (w/w) water and 2% (w/w) sodium hydroxide, > 99% reduction of 1,3‐PDO esters was achieved. This step conveniently provided free 1,3‐PDO and the sodium salt of organic acids from the corresponding esters, which increased the 1,3‐PDO yield by 7% and prevented a renewed formation of esters. After a single stage distillation from the hydrolyzed broth and a followed active carbon treatment, 1,3‐PDO with a purity of 99.63% and an overall recovery yield of 76% was obtained. No wastewater with high‐salt content was produced during the whole downstream process. The results demonstrated that the monitoring and complete elimination of 1,3‐PDO esters are crucial for the efficient separation of highly pure 1,3‐PDO with acceptable yield from fermentation broth of raw glycerol.

**Abbreviations**
1,3‐PDO: 1,3‐propanediol
GC‐MS: gas chromatography/mass spectrometry
HPLC: high performance liquid chromatography

## 1 INTRODUCTION

1,3‐Propanediol (1,3‐PDO) is an important monomer mainly used in the production of polytrimethylene terephthalate (PTT), which is a promising polymer suitable for textile and fiber manufacturing [1, 2]. Another increasing demand for 1,3‐PDO is owing to its use as a mild solvent with superior moisturizing and antimicrobial effects in various cosmetic products [3, 4]. Biosynthesis of 1,3‐PDO by fermentation provides significant advantages over traditional chemical processes, which are based on ethylene or propylene using costly catalysts and under extreme reaction conditions such as high temperature and pressure. In particular, fermentation of glycerol using clostridia bacteria under unsterile conditions strongly reduces the investment and operation costs, which makes the biological production of 1,3‐PDO more economically competitive to chemical processes [5, 6, 7]. However, the purity required for 1,3‐PDO in its industrial applications is usually higher than 99.5%, and this requires high efficient downstream processing for the commercial bio‐production of 1,3‐PDO. It is well known that the microbial production of 1,3‐PDO is usually accompanied by the formation of organic acids (acetic, butyric, lactic, formic, etc.). Their removal is one of the major steps of 1,3‐PDO downstream processing. It is indicated in literatures that organic acids may react with alcohols (e.g., 1,3‐PDO) to form esters as additional byproducts during the purification processes [8, 9], leading to unwanted color and odor in the final 1,3‐PDO product which is especially detrimental for its use in production of polymers and cosmetics. Furthermore, fermentation broth of 1,3‐PDO also contains a lot of inorganic mineral salts. If not removed completely, these salts will precipitate during the concentration process of the fermentation broth, leading to high energy consumption and low yield of the targeted product [10, 11]. Therefore, the complete elimination of salts and organic acids is a major issue for recovering 1,3‐PDO with high purity and high yield from microbial fermentation broth.

A wide variety of methods for the purification of biologically produced 1,3‐PDO have been reported. Among them, ion exchange, electrodialysis and two phase salting‐out extraction are most discussed for the removal of inorganic and organic impurities from fermentation broth before final distillation and separation of pure 1,3‐PDO. In a patent filed by the company DuPont it was described the use of a strong acid cation exchange resin followed by a week base anion exchange resin for the removal of salts. More than 98% of the mineral salts and ammonium salts of organic acids were efficiently removed. 1,3‐PDO with purity higher than 99.5% was then obtained by distillation [12]. The major shortage of this process is the quick saturation of the resins, which requires a large amount of NaOH and HCl solution for their regeneration [10, 13]. Gong et al. reported the potential use of electrodialysis for desalination before evaporation [14, 15]. Ninety percent of the organic acid salts were removed, and the loss of 1,3‐PDO was about 6% due to the diffusion through ion exchange membrane. The longer the desalination process took, the greater the loss of 1,3‐PDO was observed [16]. Wu et al. proposed to use bipolar membrane electrodialysis for desalination by converting the salts into corresponding acids and bases [17]. However, the conversion rate of salts (removal of salts) was only around 85%. It should be mentioned that the energy and material costs for electrodialysis are normally very high, prohibiting its practical use in the commercial production of cheap bulk chemicals. Furthermore, desalination by both ion exchange and electrodialysis inevitably generates a large amount of wastewater (1‐3 times of the volume of the fermentation broth), placing a huge burden on the environment.

Another strategy extensively evaluated for the separation of 1,3‐PDO is two‐phase salting‐out extraction, which uses inorganic salts as salting‐out reagents and organic solvents as extractants [18, 19, 20, 21, 22]. The solubility of 1,3‐PDO in fermentation broth decreases significantly with the aid of a salting‐out reagent, forcing 1,3‐PDO to diffuse into the extractant. As a result, fast and high recovery of 1,3‐PDO in the organic phase can be achieved with extremely low energy consumption and simple operation. Vivek et al. studied the separation of 1,3‐PDO from fermentation broth using a K_2_CO_3_+K_2_HPO_4_ / isopropanol salting‐out extraction system [18]. The maximum recovery yield of 1,3‐PDO in the solvent phase reached 98.27%. However, the removal of total organic acid salts (lactate and acetate) was less than 65%. Song et al. [21] proposed a two‐step salting‐out extraction strategy to separate 1,3‐PDO from lactic acid. In the first step, 92.4% 1,3‐PDO was recovered in the water‐poor isopropanol phase by adding 30% potassium carbonate, while close to 90% of lactic acid was enriched in the water‐rich salt phase. In the second step, 73.8% lactic acid was recovered from the salt phase by adding ethanol. Recently, Li et al. [22] showed a novel two‐step extraction using hydrophobic n‐butyl acetate and the acidic inorganic salt NaH_2_PO_4_. More than 96% butyric acid was efficiently separated from 1,3‐PDO in the first step extraction, and 95.5% 1,3‐PDO was then recovered by ethanol in the second step. It is obvious that the recovery and reuse of the massive amount of inorganic salts are crucial to make the salting‐out process more economically and environmentally sound for the industrial application, but in fact not yet evaluated systematically in any of the reported studies.

Other methods used to separate 1,3‐PDO from the fermentation broth include mainly preparative chromatography, fixed‐bed resin adsorption, liquid–liquid extraction and reactive extraction [23, 24, 25, 26, 27]. All these processes require the use of large quantities of organic solvents or hazardous reactants such as aldehydes. The investment and operation costs for large‐scale application are too high, and thus none of these processes were applied in the industrial production of 1,3‐PDO so far. It is well known that a complete evaluation including achievable final purity and overall yield of the targeted product, handling of wastes and cost calculations is indispensable before a developed downstream process is scaled up from lab to industry. However, validation of a complete purification process for recovering highly pure PDO from a raw glycerol‐based fermentation broth has been seldom done. Kaeding et al. [5] developed a downstream process for 1,3‐PDO purification including an ultrafiltration for biomass and protein separation, an evaporation step for concentration of fermentation broth and a two‐step rectification for final purification. The validation of the process was performed up to a minplant scale and Aspen plus simulation was applied for the estimations of process parameters. Highly pure 1,3‐PDO (>99%) was successfully obtained without generating any high‐salt wastewater. The theoretical overall yield of 1,3‐PDO in the developed downstream process was above 87% according to Aspen plus simulation. However, the overall yield of 1,3‐PDO obtained from the miniplant was only 57%, which was too low for the industrial production. The reasons for the significant difference between the experimentally achieved and calculated yield were not further clarified. In addition, raw glycerol was desalinated by the expensive electrodialysis before fermentation in order to minimize salt input and therefore salt precipitation in the downstream process. Hence, developing a complete and efficient downstream process to isolate 1,3‐PDO produced by microbial fermentation without the drawbacks discussed above remains a critical issue to be addressed.

**PRACTICAL APPLICATION**

A novel purification process for the production of 1,3‐propanediol (1,3‐PDO) was reported in this study. First, the problem of salt precipitation in the first vacuum distillation step (separating 1,3‐PDO and organic acids from salts) was overcome by using sufficient glycerol as a heavy solvent for the suspension of the salts. High yield of completely desalinated 1,3‐PDO was achieved with relatively low energy consumption and without any output of wastewater. Second, the formation of different types of 1,3‐PDO esters of organic acids was revealed for the first time, and the influence of the esters for the final purity and yield of 1,3‐PDO was investigated. The alkaline hydrolysis method applied led to complete elimination of the esters which significantly increased the yield of 1,3‐PDO in the final distillation step. The results are important for designing an industrial purification process of 1,3‐PDO from the microbial fermentation broth with higher recovery and less pollution.

In this study, an efficient and robust downstream process was developed to generate highly pure 1,3‐PDO from fermentation broth of raw glycerol fermentation by *Clostridium pasteurianum*. The process is composed of the following steps: (1) removal of biomass and proteins by ultrafiltration, (2) removal of water, inorganic salts, and organic acids by vacuum distillation while supplying glycerol as heavy solvent to circumvent the problem of salts precipitation, (3) alkaline hydrolysis of 1,3‐PDO esters formed during the vacuum distillation step, (4) final distillation of 1,3‐PDO, followed by deodorization with active carbon. In particular, the formation and elimination of 1,3‐PDO esters are reported for the first time and represent an important step and novelty for achieving highly pure 1,3‐PDO with satisfactory yield from fermentation of raw glycerol.

## 2 MATERIALS AND METHODS

### 2.1 Chemicals

Raw glycerol (comprising in weight percentage: glycerol 80, water 14.41, ash 3.9, matter organic non‐glycerol 2.21, salts 3.54) was kindly provided by PT Musim Mas (Indonesia). Standards of 1,3‐PDO, acetic acid and butyric acid were purchased from Sigma–Aldrich (Germany). All other chemicals used in this study were analytical grade and purchased from Carl Roth (Germany).

### 2.2 Microorganism and fermentation process

A wild‐type strain of *C. pasteurianum* which was isolated from an active sludge sample by Kaeding et al. [5] was used for the fermentation. The strain was stored at ‐80°C in 2‐mL cryo vials containing 75% active culture and 25% of glycerol (v/v). Fed‐batch fermentations were performed in a 2‐L glass bioreactor (Eppendorf, Germany) filled with 1 L fermentation medium. A synthetic medium modified from Biebl [28] was used in this study, in which biotin and calcium pantothenate (both 0.1 mg/L) were used to replace the yeast extract. A two‐step pre‐culture procedure (first overnight and second 12 h at 35°C) was adopted to prepare in anaerobic bottles. 100 mL of the second pre‐culture in exponential phase was used for inoculating 1 L of the fresh but unsterilized fermentation medium. To achieve anaerobic conditions, the medium was degassed with 0.6 vvm N_2_ for 30 min before the inoculation, and the N_2_ sparging was immediately stopped after the inoculation. Agitation speed and temperature were kept at 200 rpm and 35°C, respectively. pH was controlled at 6.5 by the automatic adjustment with 25% ammonia solution. The initial raw glycerol concentrations in the fermentation medium was 80 g/L. Continuous feeding with diluted and micro‐filtrated raw glycerol (50%, w/w) was initiated when the glycerol concentration in the medium was lower than 20 g/L. The feeding rate was set at 40 g/h, and the duration of glycerol feeding was 6 h for each fed‐batch fermentation.

### 2.3 Ultrafiltration and concentration of 1,3‐PDO fermentation broth

Direct ultrafiltration of fermentation broth for removing biomass and proteins was performed by using a hollow‐fiber membrane module (Asahi‐Kasei, Japan) made of polyacrylonitrile (inner diameter 0.8 mm, membrane area 0.19 m^2^, molecular weight cut‐off (MWCO) 1  and 6 kDa, respectively). The feed flow rate of the fermentation broth was maintained at 12 L/min, producing thereby 0.1–0.15 L/min of permeation rate and 1.5–2 bar of trans‐membrane pressure.

The permeate from ultrafiltration was transferred into a rotary evaporator to remove water. Water evaporation was performed at 80°C and under a vacuum of 200–500 mbar.

The content as weight fraction in percentage (wt%) of each component (*C*) in each purification step was calculated from Equation (1):

(1) $$C\;=\frac{c\times d\times v}{w}\;\times 100\%$$

where *c* is the concentration (g/L) determined by high performance liquid chromatography (HPLC), *d* the dilution factor, *v* the volume (L) and *w* the weight (g) of the sample taken for analysis.

The recovery (*R_d_*, recovery in the distillate, and *Rr*, recovery in the residue) and material balance (*M*) of components in the distillation process were calculated by Equations (2)–(4):

(2) $$\;R_{d}=\frac{C_{d}\times W_{d}}{C_{f}\times W_{f}}\;\times 100\%$$

(3) $$R_{r}=\frac{C_{r}\times W_{r}}{C_{f}\times W_{f}}\;\times 100\%$$

(4) $$M\;=R_{d}\;+R_{f}$$

where *C_d_*, *C_r_* and *C_f_* are the content (wt%) of the component in the distillate, residue and feed, respectively, *W_d_*, *W_r_* and *W_f_* are the total weight (g) of the distillate, residue and feed, respectively.

### 2.4 Removal of salts and organic acids

To evaluate the desalination effect by vacuum distillation, three lots of material were prepared by (1) adding raw glycerol (80 wt%) to the concentrated broth from step 2.3 to reach a glycerol content of 10 wt%, and (2) similar to (1) but with glycerol content of 15 wt%, (3) adding raw glycerol to 20 wt% and adjust the pH to 12 with 50% NaOH. Each lot was fed to the rotary evaporator operating at a vacuum of 20 mbar and a temperature of 140–180°C. Water, free organic acids and 1,3‐PDO were evaporated and collected in the distillate. Crystallized salts and heavy impurities were suspended by glycerol and collected as retentate at the flask bottom.

The distillate (desalinated broth) was then fed to the rotary evaporator operating at a vacuum of 20 mbar and a temperature of 120°C. All water and most part of the organic acids were evaporated and collected in the distillate, and a raw 1,3‐PDO product was obtained as retentate at the flask bottom.

### 2.5 Preparation of synthetic standard mixtures comprising 1,3‐PDO esters of acetic acid and butyric acid

1,3‐PDO monoesters and diesters of acetic acid and butyric acid, as well as mixed diester of acetic acid/butyric acid might be formed during the distillation process. Since no chemical standards of these esters are commercially available, three synthetic standard mixtures were prepared by mixing (1) 1,3‐PDO and acetic acid (20:80, w/w), (2) 1,3‐PDO and butyric acid (20:80, w/w), (3) 1,3‐PDO, acetic acid and butyric acid (40:30:30, w/w/w), and then allowing esterification in the oven at 55°C overnight. The formation of the five 1,3‐PDO esters mentioned above were then confirmed via gas chromatography/mass spectrometry (GC‐MS). Gas chromatography/flame ionization detection was used for the routine monitoring of the formation of the 1,3‐PDO esters.

### 2.6 Hydrolysis of 1,3‐PDO esters

Based on the determination of 1,3‐PDO esters formed, an appropriate amount of NaOH was added along with water to the raw 1,3‐PDO product obtained in step 2.4. The mixture was vortexed until NaOH was completely dissolved. Hydrolysis was carried out by stirring and heating the mixture at 60°C for 1 h. Water content in the mixture was fixed at 20% (w/w), and different contents (0.5, 1 and 2 wt%) of NaOH were tested. After the hydrolysis treatment, water was completely removed by vacuum distillation operating at a vacuum of 100 mbar and a temperature of 100°C. The hydrolysis efficiency of 1,3‐PDO esters was monitored by GC/FID analysis.

### 2.7 Analytical methods

The biomass concentration was measured as optical density at 600 nm with a Genesys 10 UV Scanning photometer (Thermo Scientific, Germany) using 1‐mm cuvettes. For samples with optical density (OD) > 0.8, the sample was diluted with milli‐Q water.

The concentration of protein was measured by the Coomassie brilliant blue method using bovine serum albumin (BSA) as the standard protein [29].

The concentrations of glycerol, 1,3‐PDO, acetate, and butyrate were measured by HPLC (Kontron Instruments, Switzerland), with a 5‐mM sulfuric acid as the eluent at a flow rate of 0.6 mL/min, and an Aminex HPX‐87H 300 mm x 7.8 mm column (Bio‐Rad Laboratories, USA) as the separation column at an operation temperature of 30°C, The column was successively coupled to a UV‐detector (Shimadzu, Japan) at a wavelength of 210 nm and a differential refractometer RI‐detector (Kontron Instruments, Switzerland).

GC‐MS analysis was carried out on an Agilent 7890B gas chromatograph coupled to an Agilent MSD 5977A mass spectrometer. The GC column used was a DB‐624 ultra‐inert capillary column (30 m x 0.250 mm, 1.4 μm film thickness). 1 μL of each sample were injected at a split ratio of 1:5 to the injector operating at 250°C. Helium was used as the carrier gas at a column flow rate of 1 mL/min. The column oven program was: initial temperature 70°C (hold for 2 min), ramp at 10°C/min to 220°C (hold for 15 min). The mass selective detector was operated in scan mode in the range of 2–500 amu. The mass selective detector transfer line was set at 280°C and the temperature of the MS Quad and MS Source was set at 150°C and 230°C, respectively. Identification was done by searching the acquired MS spectra against the NIST database.

GC analysis was performed on a phenomenex HP‐5 capillary column (30 m x 0.25 mm, 0.25 μm film thickness) using a Varian 3900 GC instrument equipped with a flame ionization detector (FID). 0.5 μL of each sample were injected at a split ratio of 1:10 to the injector operating at 250°C. Nitrogen was used as the carrier gas at a column flow rate of 1.5 mL/min. The column oven temperature program used was: initial temperature 70°C (hold for 2 min), then ramp to 220°C at 10°C/min (hold for 5 min). The FID detector was operated at 300°C.

Element analysis by inductively coupled plasma‐optical emission spectroscopy (ICP‐OES) or inductively coupled plasma‐mass spectrometry (ICP‐MS) were carried out as follows: approximately 500 mg of each sample was first mixed with 2 mL of MilliQ water and then added with 5 mL of concentrated HNO_3_, and left to stand for about 5 min, followed by adding 2 mL of 30% H_2_O_2_ solution to increase the oxidizing effect. Then, samples were slowly heated to 180°C and held at 180°C for 10 min. After cooling, samples were diluted with MilliQ water to a final volume of 14 mL. The contents of Na, K, Mg, Ca, Fe, P and S were determined by ICP‐OES on a Perkin Elmer Optima 8300 DV ICP‐OES in the concentration range of μg/L to mg/L, or by ICP‐MS on a Perkin Elmer NexION 300 D ICP‐MS in the low μg/L level. For total nitrogen (TN) determination each sample was simply diluted in a ratio of 1 to 50 with MilliQ water before analyzed on a Multi N/C 3100 Analyzer (Analytic Jena GmbH, Jena, Germany).

## 3 RESULTS AND DISCUSSION

### 3.1 1,3‐PDO fermentation broth and its pretreatment

**FIGURE 1:**
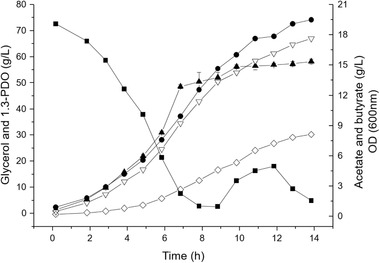
Fed‐batch fermentation of raw glycerol with *C. pasteurianum* under unsterile conditions in a synthetic medium without yeast extract. (■) glycerol, (●) 1,3‐PDO, (**▽**) acetate, (◇) butyrate, and (▲) OD. The OD value is the average from three independent measurements, and the values of glycerol and the products are the average values from two independent measurements

Fed‐batch fermentations of raw glycerol using *C. pasteurianum* were carried out under unsterile conditions in a synthetic medium without yeast extract. The results of a typical fermentation are shown in Figure 1. 1,3‐PDO concentration as high as 74 g/L was achieved after a fermentation time of only 14 h with a yield of 0.52 g PDO/g glycerol and a volumetric productivity of 5.3 g/L h, representing one of the most efficient glycerol fermentation processes reported so far. 5 kg fermentation broth from 4 independent fed‐batch fermentations were mixed together and used for evaluating the downstream process. Table 1 summarizes the results from these 4 fed‐batch fermentations.

**TABLE 1:** Experimental results of 4 fed‐batch fermentations of raw glycerol to 1,3‐PDO by *C. pasteurianum*

Concentration (g/L)									
Biomass	Residue glycerol	1,3‐PDO	Acetate	Butyrate	Butanol	Lactate	Formate	Yield (g PDO/g Glycerol)	Overall PDO productivity (g/L h)
9.071±1.60	5.12±1.72	74.62±0.31	16.48±1.75	10.00±1.64	1.29±1.07	0.26±0.26	0.62±0.14	0.52±0.02	5.02±0.29

First, ultrafiltration was carried out to remove biomass and proteins from the culture broth. Two types of hollow fiber membrane with the same inner diameter but different molecular weight cut‐offs (MWCO of 1 and 6 kDa, respectively) were tested (Table 2). Both ultrafiltration membranes successfully removed more than 99% of the cells and proteins. Colored impurities were more efficiently removed using the 1 kDa membrane, which gave a completely colorless permeate. However, the concentration of 1,3‐PDO also decreased by 25% which was much higher than that (0.8%) with the 6 kDa membrane. This was due to a quick fouling of the 1 kDa membrane. Under the condition of maintaining the same feeding rate, the pressure of 6 kDa membrane maintained at 1,5‐2 bar during the whole process of filtrating 2 L of the fermentation broth, and the permeation rate maintained at 47 L/(m^2^ h). On the contrary, the pressure of the 1 kDa membrane increased to 5 bar and the permeation rate already decreased to less than 32 L/(m^2^ h) after 1 L of the fermentation broth was filtrated. This indicates that when ultrafiltration with low MWCO membrane or even nanofiltration is to be used, removal of biomass by microfiltration or centrifugation beforehand might be necessary. Given that the biomass and proteins can be removed simultaneously, and the total loss of 1,3‐PDO was less than 5% (mainly lost in the residue retentate), direct ultrafiltration of fermentation broth using the 6 kDa membrane is an appropriate choice as the first step of 1,3‐PDO purification.

**TABLE 2:** Comparison of direct ultrafiltration of fermentation broth with membranes of two different MWCO

	Biomass (OD_600_)	Protein (g/L)	1,3‐PDO (g/L)	Color
Initial broth	15.36	0.26	74.20	Brown
Permeate (MWCO 6 kDa)	0.043	<0.10	73.60	Yellow
Permeate (MWCO 1 kDa)	0.035	<0.10	55.80	Colorless

The permeate obtained from the ultrafiltration with the 6 kDa membrane was further concentrated in a rotary evaporator at 80°C and under a vacuum of 200–500 mbar, resulting thereby a broth with the weight being reduced by 85%. The composition of the concentrated broth was 1,3‐PDO (47.3 wt%), glycerol (0.75 wt%), acetate (10.1 wt%), butyrate (4.8 wt%), water and residue medium components. The weight percentage losses of 1,3‐PDO, glycerol, acetate and butyrate due to entrainment by the evaporated water were 1.6%, 0, 6.5% and 12.6%, respectively. No strong foaming was observed during the evaporation process, indicating a sufficient protein removal by the ultrafiltration from the fermentation broth, as also described by Kaeding et al. [5].

### 3.2 Removal of salts and organic acids

**FIGURE 2:**
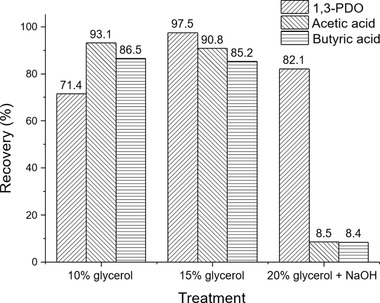
Recovery of 1,3‐PDO, acetic acid and butyric acid from the salts removal step. Distillation conditions: temperature 140–180°C and vacuum pressure 20 mbar

By the vacuum distillation strategy employed in this study, a raw 1,3‐PDO product can be evaporated as distillate and separated from high boiling salts without generating any high‐salts wastewater. However, crystallization of salts in the fermentation broth happened during the distillation process, and the deposition of the crystallized salts resulted in a viscous slurry at the flask bottom that led to high energy consumption and low yield of the target product [10]. In order to improve the distillation efficiency, the effect of glycerol as a heavy solvent for dispersing the salts was studied. To this end, the concentrated broth obtained after ultrafiltration and water evaporation was supplemented with raw glycerol at two different weight fractions, namely 10 and 15 wt%, and then fed into a rotary evaporator for the distillative separation of 1,3‐PDO from salts. As shown in Figure 2, the recovery of 1,3‐PDO in the distillate from the feed containing 15 wt% glycerol was much higher than that from the feed containing only 10 wt% glycerol (97.5% vs. 71.4%). The fast rotation of the evaporating flask generated a continuous flow of glycerol which drove the crystallized salts to keep moving in the flask. Better fluidity of the residue left at the bottom was achieved with higher glycerol content, and more than 80 wt% of the feed was evaporated at a temperature of 140°C and a vacuum of 20 mbar. In contrast, residue resulted from the feed containing less glycerol was more viscous, and only 65 wt% of the feed was evaporated under the same operating conditions. Increasing temperature to 150 and 160°C improved the recovery of 1,3‐PDO from 71.4% to 81.9% and 84.1%, respectively. However, it consumed more energy and the yield was still relatively low. The efficiency of salt elimination was determined through element analysis of the distillate using ICP‐OES and ICP‐MS and demonstrated that more than 98% of the salts were removed in this step (Table 3). Although a higher content of glycerol contributes to a better recovery of 1,3‐PDO in the salt elimination step by vacuum distillation, the cost of glycerol needs to be considered for the downstream process. Glycerol and salts can be easily separated by adding ethanol to the residue to provoke precipitation of the salts. After filtration the ethanol solution was evaporated under reduced pressure to recycle the ethanol, and a dark yellow but transparent liquid containing more than 90% of glycerol was obtained. The recycled “raw glycerol” can be theoretically reused in the salts removal step, but yet needs to be further tested. In the industrial process, thin film evaporator equipped with an inner wall scraper can be used to generate a continuous flow of glycerol for the suspension of salts during the distillation process [11].

**TABLE 3:** Quantitative elemental analysis of the products from different purification steps

	Concentration (mg/L)			
Elements	Concentrated broth	Product from salts removal	1,3‐PDO before active carbon treatment	1,3‐PDO after active carbon treatment
Na	819	<13.4	<13.4	<13.4
K	813	<11.2	<11.2	<11.2
Ca	627	<0.28	<0.28	<0.28
Mg	77	<0.56	<0.56	<0.56
Fe	11	<0.56	<0.56	<0.56
N	33400	27700	280	190
S	2140	45.4	12.8	7.8
P	457	<2.8	<2.8	<2.8

In the next step, the distillate from the salt removal step was fed into a rotary evaporator to further remove the organic acids by vacuum distillation (120°C, vacuum 20 mbar). 71.6 wt% acetic acid and 55.6 wt% butyric acid were removed to the distillate, along with 12.5 wt% 1,3‐PDO loss. Nevertheless, the content of 1,3‐PDO in the retentate increased from 50.4 to 79.8 wt% accordingly. However, the weight fraction of 1,3‐PDO, acetic acid and butyric acid remained in the retentate was only 80.1%, 10.7% and 20.7%, respectively, which resulted in a material balance significantly less than 100% for each of them (Table 4).

**TABLE 4:** Content, recovery and mass balance calculations of 1,3‐PDO, acetate and butyrate in each purification step

		1,3‐PDO			Acetic acid			Butyric acid		
Steps	Mass (g)	Content (%)	Recovery (%)	Balance (%)	Content (%)	Recovery (%)	Balance (%)	Content (%)	Recovery (%)	Balance (%)
Ultrafiltration										
Feed	5000	7.6	–	99.6	1.8	–	97.8	0.80	–	108
Residue	200	9.3	4.9		3.2	7.1		0	0	
Permeate	4800	7.5	94.7		1.7	90.7		0.90	108	
Concentration										
Feed	4800	7.5	–	97.5	1.7	–	96.9	0.90	–	93.7
Residue	730	47.3	95.9		10.1	90.4		4.8	81.1	
Distillate	4070	0.14	1.6		0.13	6.5		0.13	12.6	
Salt removal										
Feed + glycerol	400	44.1	–	99.2	9.2	–	95.9	4.5	–	96.9
Residue	59	5.2	1.7		3.2	5.1		3.6	11.7	
Distillate	341	50.4	97.5		9.8	90.8		4.5	85.2	
Acid removal										
Feed	216	50.4	–	92.6	9.8	–	82.2	4.5	–	76.3
Residue	109	79.8	80.1		2.1	10.7		1.8	20.7	
Distillate	107	12.8	12.5		14.2	71.6		5.1	55.6	
Hydrolysis										
Feed	109	79.8	–	–	2.1	–		1.8	–	–
Product after hydrolysis and dewatering	110	86.9	109.9		2.6	124.9		2.2	123.3	
PDO distillation										
Feed	110	86.9	–	98.6	2.6	–	92.8	2.2	–	94.7
Residue	19.1	19.0	3.8		13.9	92.8		12.0	94.7	
Distillate	90.9	99.3	94.8		0	0		0	0	
Deodorization										
Feed	90	99.3	–	–	0	–	–	0	–	–
Product after active carbon treatment	89	99.6	99.2		0	–		0	–	

It was reported that distillation of 1,3‐PDO under acidic conditions may lead to the formation of by‐products and color. 1,3‐PDO isolated and purified under such conditions is not suitable for producing good quality and color‐free polyester [8, 12]. In fact, the pH of the concentrated broth from water evaporation was reduced from 6.5 to 2 after the salt removal step. It seemed that ammonium salts of organic acids were liberated as ammonia gas and free organic acids under the vacuum heating at 140°C, leading to reduced pH. In the subsequent acid removal step, a part of 1,3‐PDO and the free organic acids might have undergone esterification reactions under the given distillation conditions, resulting in the material imbalance mentioned above. The missing parts of 1,3‐PDO, acetic acid and butyric acid for 100% material balance were 105.5, 62.7 and 26.1 mmol, respectively, based on the experimental data shown in Table 4. The ratio of the missing 1,3‐PDO to the missing organic acids is 1:0.85, which is very close to 1:1. Therefore, it was assumed that monoesters of 1,3‐PDO‐acetic acid and 1,3‐PDO‐butyric acid were the main impurities formed in the acid removal step.

To overcome this problem, the concentrated broth from water evaporation was added with 50% NaOH to increase the pH to 12 prior to the distillation for salt removal. The rationale behind it is that compared to ammonium salts, sodium or potassium salts of organic acids are much more stable under heating [5]. The presence of acetic acid and butyric acid in dissociated forms should prevent their esterification with 1,3‐PDO during the distillation. The amount of supplemented glycerol was also increased to 20 wt%, since the additionally formed sodium salts of organic acids were expected to precipitate during the distillation. As shown in Figure 2, the benefit of the alkalization was demonstrated by the removal of more than 90% of the organic acids together with the mineral salts. However, it was not until the heating temperature reached 180°C when the evaporation of 1,3‐PDO stopped, and the residue at the flask bottom became a solid wax without any fluidity, indicating reduced heat transfer efficiency and significantly higher energy consumption for 1,3‐PDO distillation. Furthermore, a small fraction of the acetic acid and butyric acid (about 8 wt% each) were still evaporated together with 1,3‐PDO. Thus, considering the high cost for energy, glycerol and NaOH and the high loss of 1,3‐PDO, the elimination of organic acids as sodium salts by adding large quantities of sodium base is unsuitable for the industrial process.

### 3.3 Identification and hydrolysis of 1,3‐PDO esters

**FIGURE 3:**
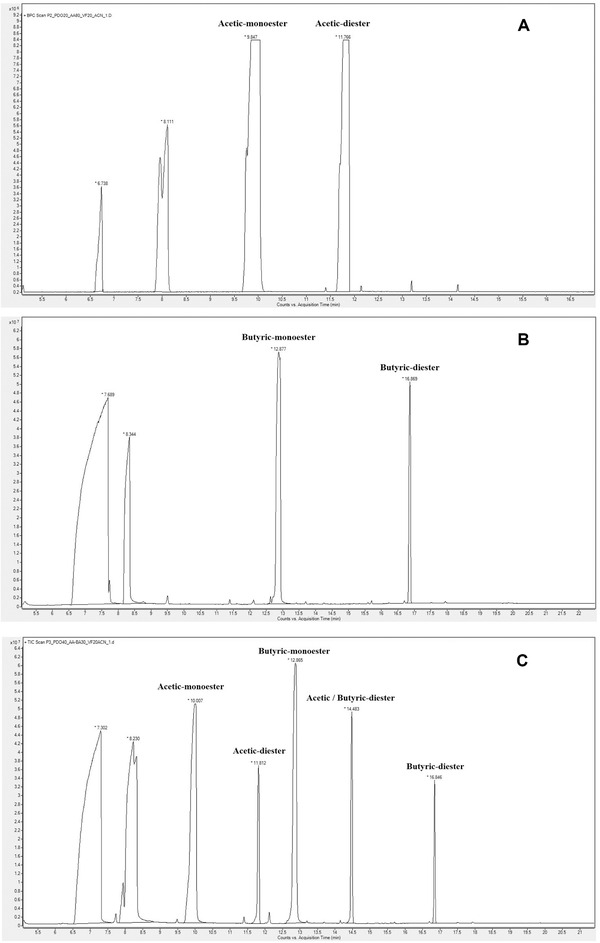
TIC chromatograms of the synthetic mixtures (A‐C) containing 1,3‐PDO mono and diesters of acetic acid and butyric acid. (A) 1,3‐PDO and acetic acid (20:80, w/w), (B) 1,3‐PDO and butyric acid (20:80, w/w), (C) 1,3‐PDO, acetic acid and butyric acid (40:30:30, w/w/w). Each sample was diluted with acetonitrile at a ratio of 1 to 20 before GC/MS analysis. Only 1,3‐PDO diester of acetic acid (1,3‐PDO diacetate) was found in the NIST database, the other four 1,3‐PDO esters were identified based on their retention times and characteristic MS fragmentations

In order to confirm whether 1,3‐PDO monoesters and diesters of acetic acid and butyric acid, as well as the mixed diester of acetic acid/butyric acid were formed during the acids removal step, GC and GC‐MS analysis were performed for the identification and monitoring of the formation of these esters. Since there are no chemical standards commercially available for the five targeted esters, synthetic standard mixtures were prepared from esterification of pure 1,3‐PDO with acetic acid and butyric acid, respectively, and with a mixture of both acids. It is observed from the GC/MS results that the five ester species were all formed expectedly based on the retention time of the five peaks on total ion chromatographs (TIC) (Figure 3A‐C) and the corresponding mass spectra shown their fragmentation information (Figure S1A‐E). The same synthetic standard samples were also run by GC/FID to establish a method for the routine monitoring of the five ester species to evaluate the elimination efficiency of these ester impurities in the following purification process (Figure S2A‐C).

**FIGURE 4:**
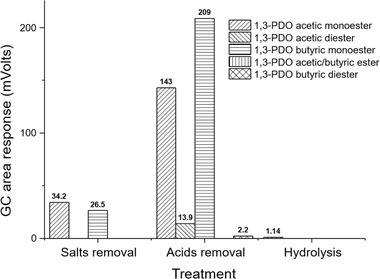
GC monitoring of the formation and elimination of 1,3‐PDO esters in different purification steps

As shown in Figure 4, a small amount of 1,3‐PDO monoesters of acetic acid and butyric acid were formed in the salts removal step. No formation of diesters and the mixed ester was observed in the desalinated sample. After the acids removal step, the formation of both monoesters increased dramatically, where the peak area increased 4.4 times for the acetic monoester, and 7.8 times in the case of the butyric monoester. In addition, a small amount of diesters were also found in the sample. However, no formation of the acetic/butyric mixed ester was observed throughout the purification process. These results strongly confirmed our previous assumption that the monoesters were the main esterification products and therefore the main reason for the unsatisfying material balance.

Of course, these esters must be removed from the 1,3‐PDO product. First of all, separation of 1,3‐PDO and the esters were tried directly by vacuum distillation. 123 g of the product after salt removal and acid removal steps described above were subjected to the rotary evaporator at a temperature of 140°C and a vacuum of 20 mbar. Three distillate fractions with the weight of 10.4, 19.7 and 59.4 g were collected successively, and subsequently detected by HPLC and GC. As shown in Table 5, evaporation of 1,3‐PDO was accompanied by a large amount of the monoesters of acetic acid and butyric acid in all three distillate fractions, indicating that 1,3‐PDO and the esters may have boiling points very close to each other, and they cannot be completely separated by a single stage distillation. Pascal Rousseaux and Clement Cellier in their patent described the rectification of concentrated and desalinated fermentation broth containing 1,3‐PDO and organic acids. Although a fraction of pure 1,3‐PDO (>99.5% by HPLC) was obtained from a two‐step batch vacuum distillation using a multi‐stage column and high reflux ratio, the yield of 1,3‐PDO in the rectification step was only 68.8% [11]. Kaeding et al. also described a similar rectification process and achieved a yield of about 71% of purified 1,3‐PDO (> 99%), although the simulation by Aspen Plus gave an excellent theoretical yield of 98% [5]. The discrepancy might be due to the fact that the simulation process did not take the formation of 1,3‐PDO esters into consideration. Therefore, in order to obtain highly pure 1,3‐PDO with acceptable yield, it is necessary to completely eliminate the formed 1,3‐PDO esters before conducting the final rectification process.

**TABLE 5:** Separation of 1,3‐PDO and its acetic and butyric esters by single stage distillation

Distillate	1,3‐PDO (%)	Acetic acid (%)	Butyric acid (%)	1,3‐PDO‐acetic monoester (peak area in mV)	1,3‐PDO‐butyric monoester (peak area in mV)
1	64.8	8.9	11.5	1445	317
2	70.4	2.1	7.2	441	829
3	89.8	0.1	1.1	396	178

Alkaline hydrolysis using NaOH as catalyst is an efficient and simple strategy for the elimination of the 1,3‐PDO esters which should result in 1,3‐PDO and the corresponding sodium carboxylic acids. Therefore, raw product obtained from acids removal step was subjected to alkaline hydrolysis. As shown in Figure 4 and Table 4, after the hydrolysis with 20 wt% water and 2 wt% NaOH at 60°C for 1 h, and subsequent dewatering by vacuum distillation, more than 99% of the ester species were successfully eliminated. Only a very small GC peak (1.14 mV) of the 1,3‐PDO‐acetic monoester was still observed. As a result, the content of 1,3‐PDO increased from 79.8 wt% to 86.9 wt% thanks to the liberation of 1,3‐PDO from the esters. Hydrolysis with lower concentration of NaOH (0.5 and 1 wt %) was also tested. However, a considerable part of esters were still detectable by GC, which indicated insufficient hydrolysis of 1,3‐PDO esters (data not shown). The amount of NaOH required for the complete hydrolysis of ester impurities can be confirmed under the sensitive monitoring of ester peaks response by GC. Analytical methods for the absolute quantitation of different types of 1,3‐PDO esters of organic acids are to be developed in order to determine the addition of NaOH directly and precisely. It is worth mentioning that the amount of NaOH used in the hydrolysis treatment is relatively less, because most of the organic acids have been removed in the previous step.

### 3.4 Final distillation of 1,3‐PDO and deodorization by active carbon

**FIGURE 5:**
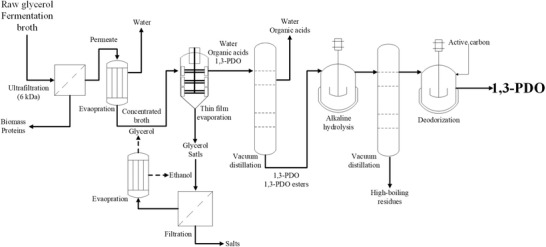
Industrial flow chat of the presented purification process for recovering 1,3‐PDO with high purity and high yield from raw glycerol‐based fermentation broth

Finally, 110 g of the product (1,3‐PDO 86.9 wt%, glycerol 4.66 wt%, sodium acetate 2.61 wt% and sodium butyrate 2.16 wt%) obtained from the hydrolysis treatment was fed to the rotary evaporator for the final distillation and separation of 1,3‐PDO at 140°C and a vacuum of 20 mbar. Totally, 90.9 g of colorless distillate was collected. The purity of 1,3‐PDO in the distillate was 99.27 wt% as measured by HPLC, with a high yield of 94.8% (Table 4). No organic acid was detected in the distillate and no 1,3‐PDO esters were formed again in the final distillation process, indicating that organic acids in undissociated form were not present after alkaline hydrolysis. However, the obtained 1,3‐PDO product still had a characteristic odor, which might be due to the contamination of some unknown volatile impurities. Therefore, the final product was deodorized via treatment with 2 wt% active carbon by stirring at room temperature for 3 h. After separation of active carbon by filtration, colorless and odorless 1,3‐PDO with a high purity of 99.63 wt% was finally obtained. It is observed that the concentration of nitrogen and sulfur in the product decreased from 280 to 190 mg/L, and from 12.8 to 7.8 mg/L, respectively, after the active carbon treatment (Table 3). This indicates that the nitrogen and sulfur containing compounds might be the main reason for the odor problem, and also needs to be eliminated efficiently to give a qualified 1,3‐PDO product. The complete downstream process for recovering 1,3‐PDO with high purity and high yield from the raw glycerol‐based fermentation broth is schematically presented in Figure 5.

## 4 CONCLUDING REMARKS

Separation and purification of highly pure 1,3‐PDO from microbial fermentation broth with complex compositions remain a major issue for industrial production of bio‐based 1,3‐PDO. This study presents a complete and novel downstream process for the recovery of 1,3‐PDO with both high purity and high yield from a raw glycerol‐based fermentation broth in lab scale. In addition to the removal of biomass and proteins by ultrafiltration, the removal of salts and organic acids are studied in more detail. In particular, vacuum distillation with continuous addition and regeneration of glycerol as a supporting agent turned out to be efficient for initial recovery of 1,3‐PDO and organic acids and efficient removal of salts from concentrated fermentation broth after water evaporation. For the first time, we observed and identified the formation of different forms of esters due to reactions of 1,3‐PDO with organic acids during the distillation process. Strategies for the hydrolytic elimination of the esters and further purification of 1,3‐PDO were proposed, resulting in successful separation of highly pure 1,3‐PDO (>99 wt%) by only using a single stage distillation apparatus. Final treatment of the product with 2 wt% active carbon resulted in a colorless and odorless 1,3‐PDO solution with a high purity of 99.63 wt%. The overall 1,3‐PDO yield of the whole process is 76%. These results provide useful information for designing a practical and feasible downstream process for the biological production of 1,3‐PDO in industrial scale.

## CONFLICT OF INTEREST

The authors have declared no conflict of interest.

## Supporting information

Supporting information.

FIGURE S1 Mass fragmentation spectra of five 1,3‐PDO esters of organic acids. A: 1,3‐PDO acetic monoester, B: 1,3‐PDO acetic diester, C: 1,3‐PDO butyric monoester, D: 1,3‐PDO acetic/butyric ester and E: 1,3‐PDO butyric diester.

FIGURE S2 GC/FID chromatograms of the synthetic mixtures containing 1,3‐PDO esters of acetic acid and butyric acid. A: 1,3‐PDO and acetic acid (20:80, w/w), B: 1,3‐PDO and butyric acid (20:80, w/w), C: 1,3‐PDO, acetic acid and butyric acid (40:30:30, w/w/w).

## Data Availability

The data that support the findings of this study are available from the corresponding author upon reasonable request.
