# Supplementary material for: A novel downstream process for highly pure 1,3‐propanediol from an efficient fed‐batch fermentation of raw glycerol by Clostridium pasteurianum
Source: Eng Life Sci. 2021 May 7;21(6):351–63. doi: 10.1002/elsc.202100012 (PMC8182277; doi:10.1002/elsc.202100012)
Supplement: Supplementary file 1 — Supporting information. [file ELSC-21-351-s001.pdf]

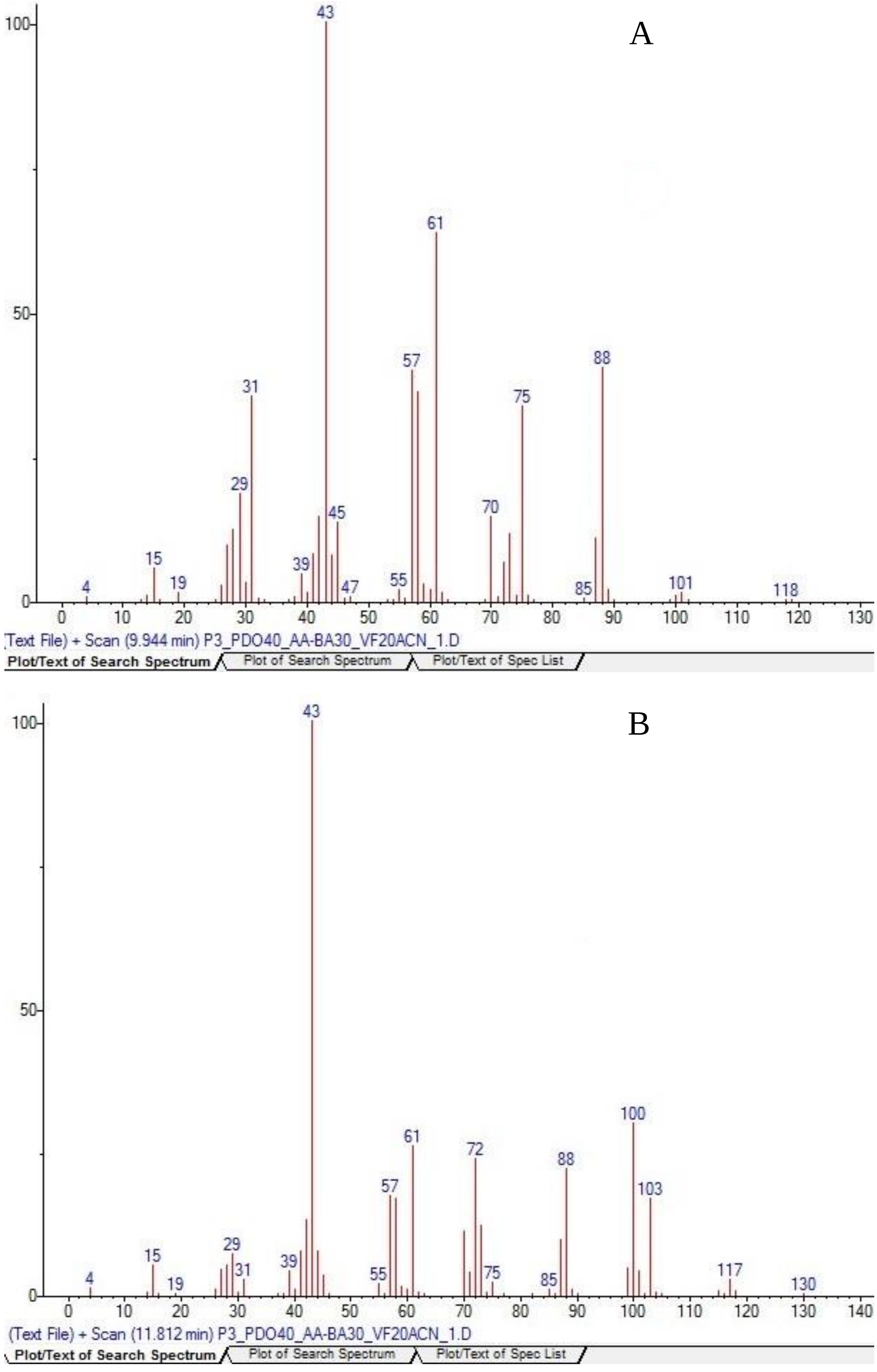

A
43
61
57
88
31
75
29
70
45
15
39
4
19
47
55
85
101
118
(Text File) + Scan (9.944 min) P3_PDO40_AA-BA30_VF20ACN_1.D
Plot/Text of Search Spectrum
Plot of Search Spectrum
Plot/Text of Spec List
B
43
100
61
72
88
57
103
29
15
31
39
4
19
55
75
85
117
130
(Text File) + Scan (11.812 min) P3_PDO40_AA-BA30_VF20ACN_1.D
Plot/Text of Search Spectrum
Plot of Search Spectrum
Plot/Text of Spec List

C

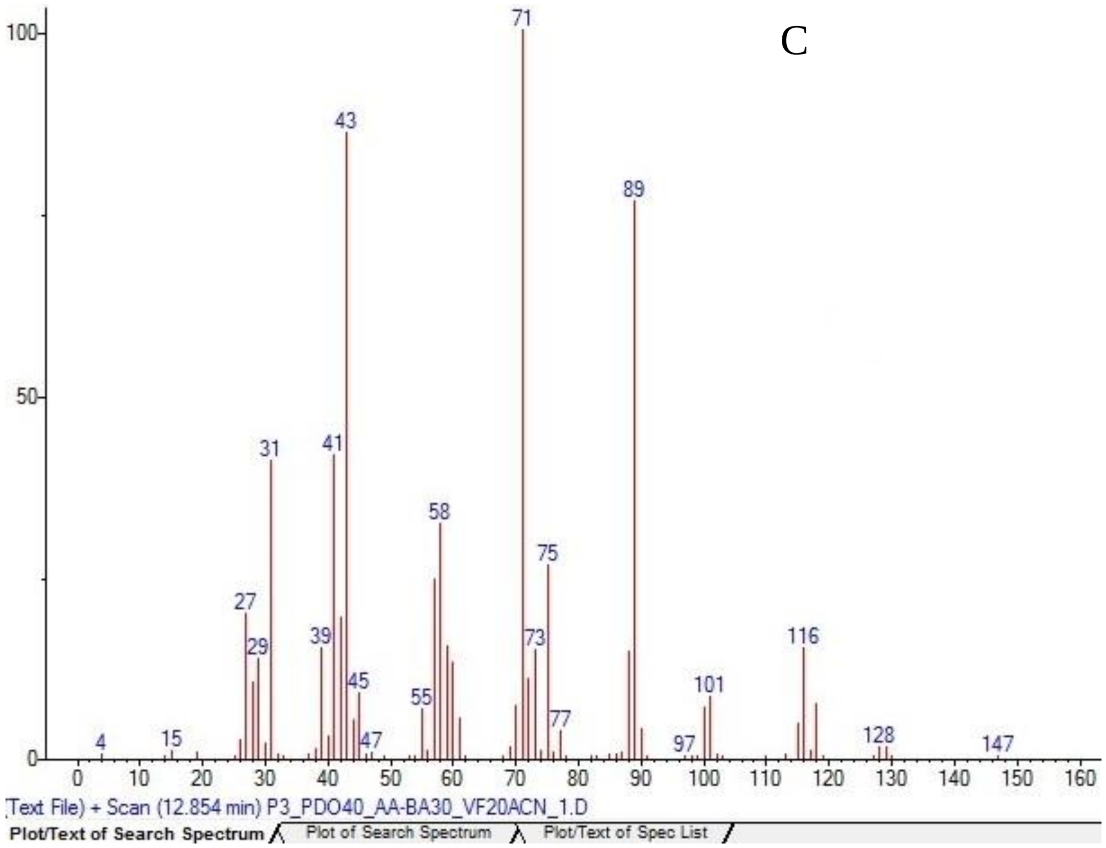

71
43
89
31
41
58
75
27
39
73
116
29
45
101
55
77
4
15
47
97
128
147
(Text File) + Scan (12.854 min) P3_PDO40_AA-BA30_VF20ACN_1.D
Plot/Text of Search Spectrum
Plot of Search Spectrum
Plot/Text of Spec List

D

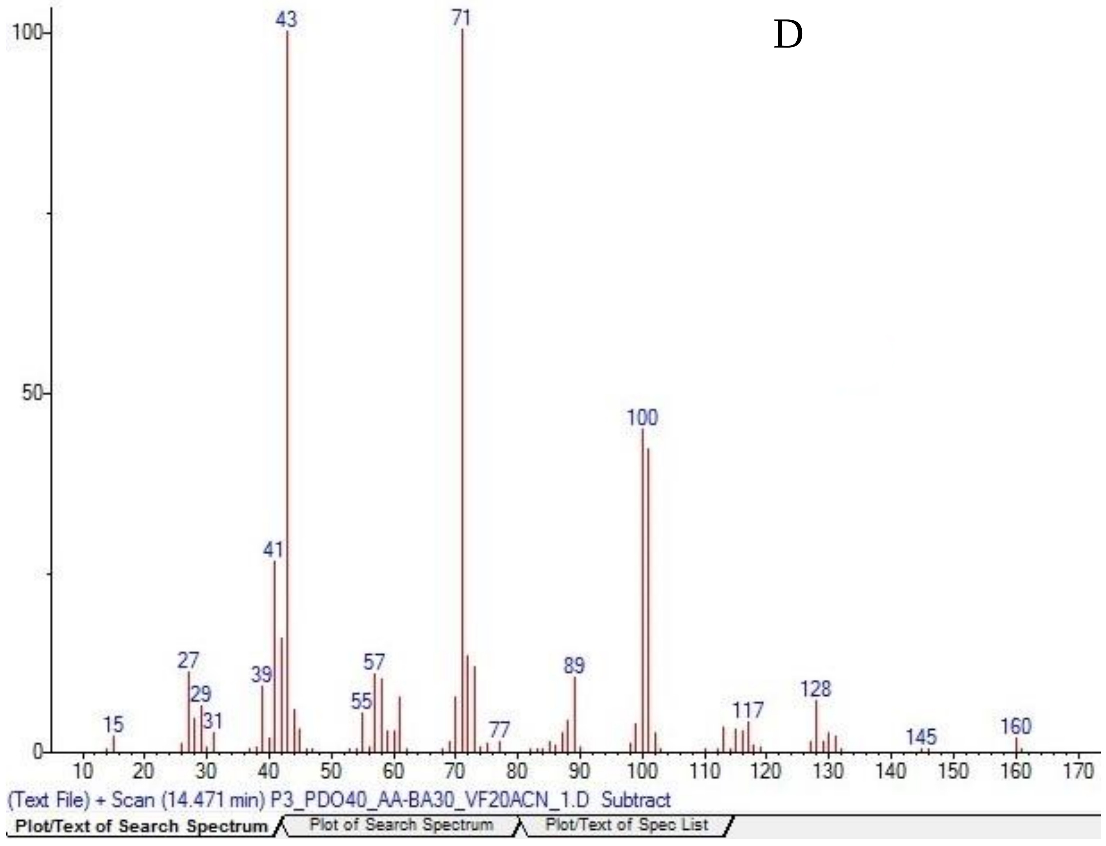

43
71
100
41
27
57
89
39
128
29
55
117
15
31
77
145
160
(Text File) + Scan (14.471 min) P3_PDO40_AA-BA30_VF20ACN_1.D Subtract
Plot/Text of Search Spectrum
Plot of Search Spectrum
Plot/Text of Spec List

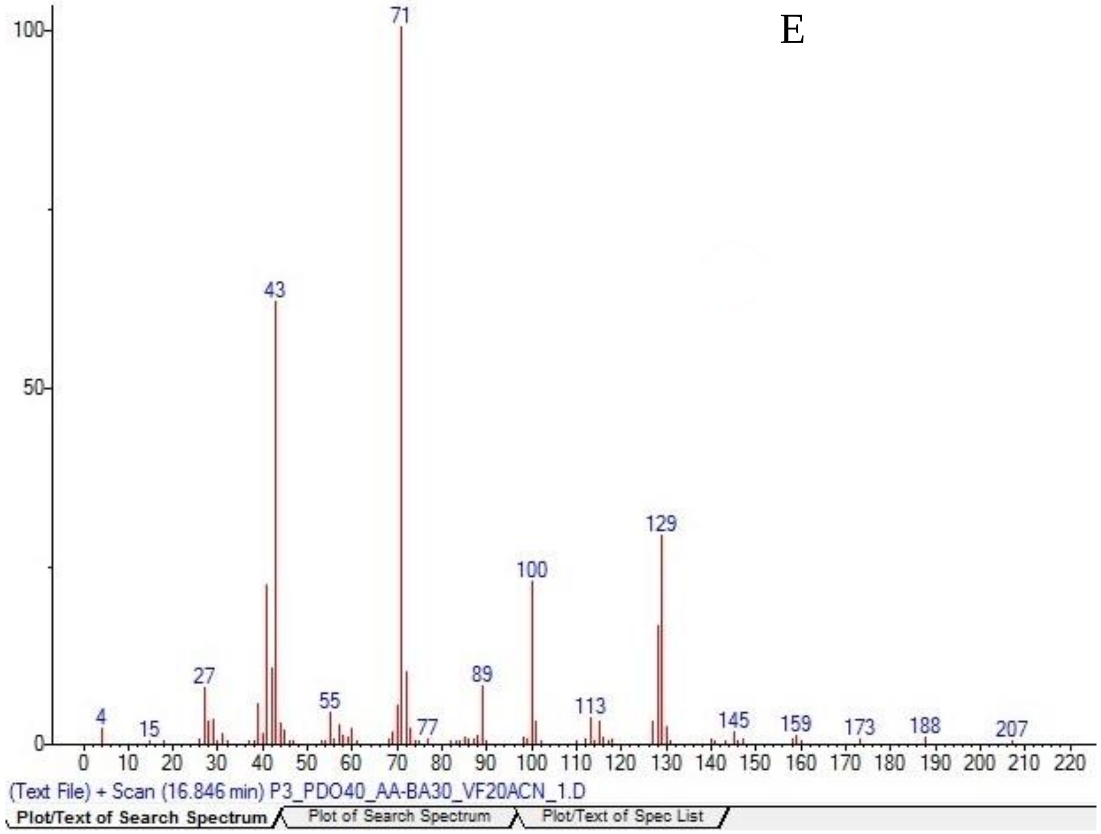

FIGURE S1 Mass fragmentation spectra of five 1,3-PDO esters of organic acids. A: 1,3-PDO acetic monoester, B: 1,3-PDO acetic diester, C: 1,3-PDO butyric monoester, D: 1,3-PDO acetic/butyric ester and E: 1,3-PDO butyric diester.

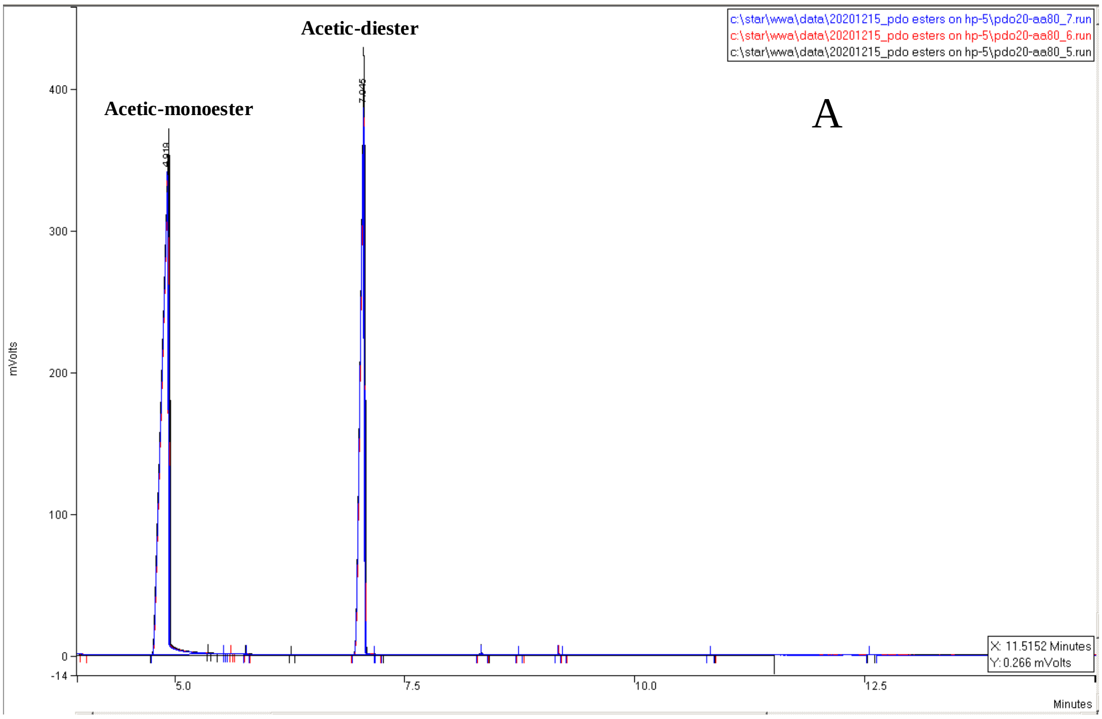

Acetic-diester
Acetic-monoester
A
c:\star\wwa\data\20201215_pdo esters on hp-5\pdo20-aa80_7.run
c:\star\wwa\data\20201215_pdo esters on hp-5\pdo20-aa80_6.run
c:\star\wwa\data\20201215_pdo esters on hp-5\pdo20-aa80_5.run
mVolts
Minutes
X: 11.5152 Minutes
Y: 0.266 mVolts

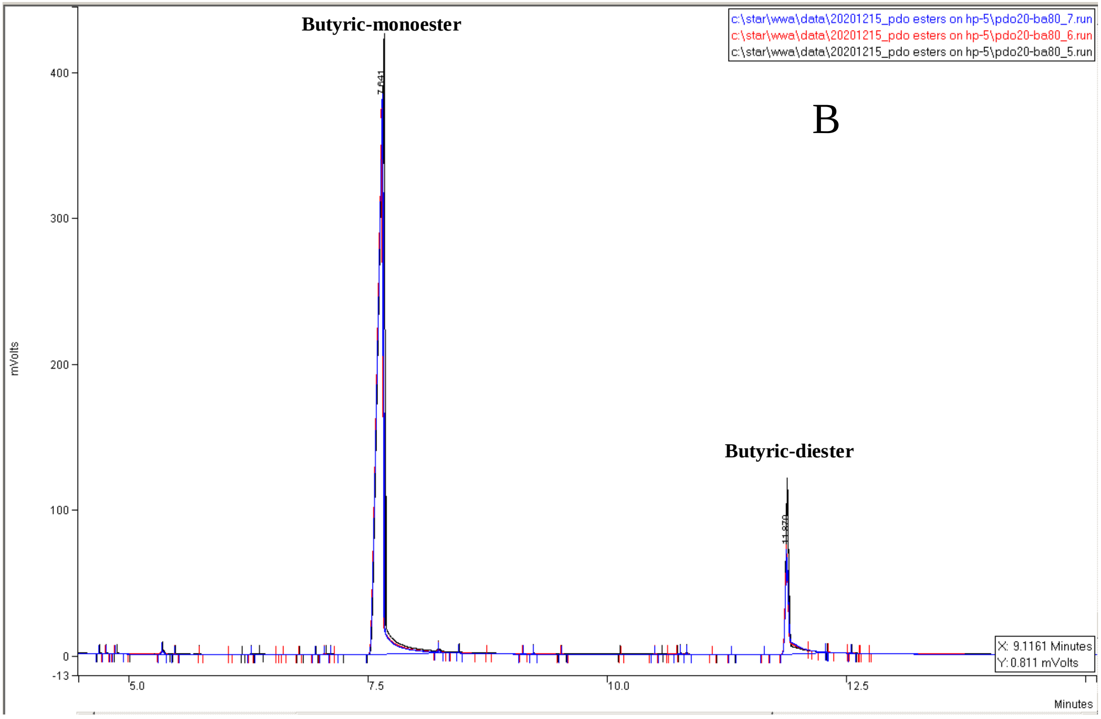

Butyric-monoester
Butyric-diester
B
c:\star\wwa\data\20201215_pdo esters on hp-5\pdo20-ba80_7.run
c:\star\wwa\data\20201215_pdo esters on hp-5\pdo20-ba80_6.run
c:\star\wwa\data\20201215_pdo esters on hp-5\pdo20-ba80_5.run
mVolts
Minutes
X: 9.1161 Minutes
Y: 0.811 mVolts

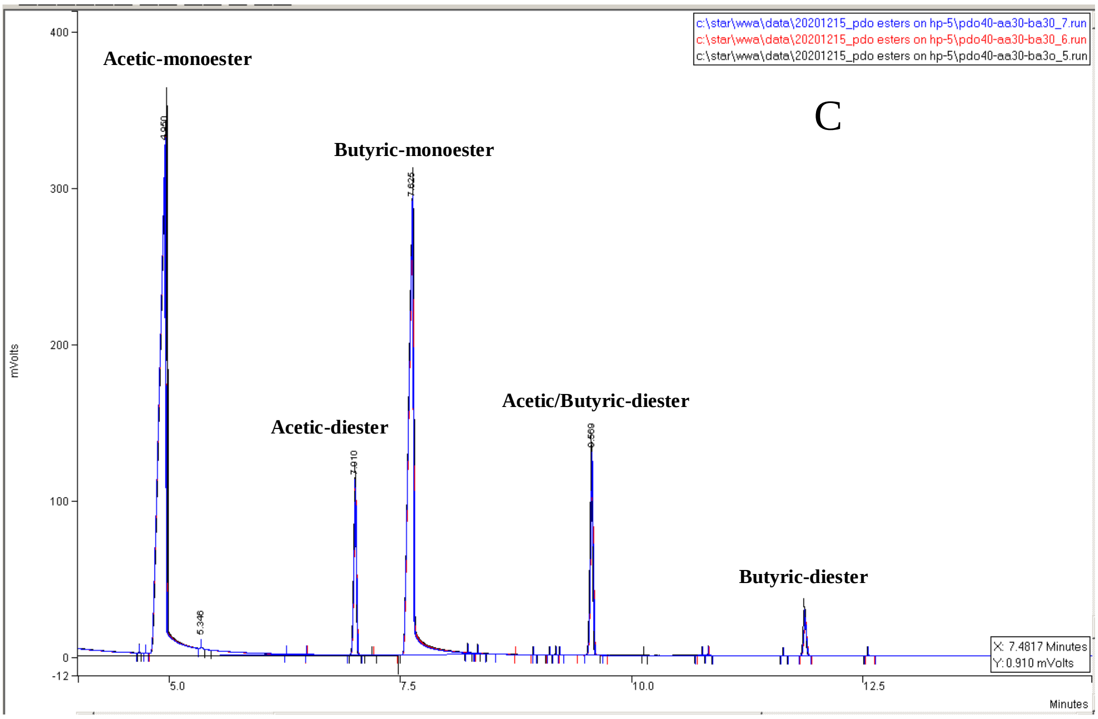

FIGURE S2 GC/FID chromatograms of the synthetic mixtures containing 1,3-PDO esters of acetic acid and butyric acid. A: 1,3-PDO and acetic acid (20:80, w/w), B: 1,3-PDO and butyric acid (20:80, w/w), C: 1,3-PDO, acetic acid and butyric acid (40:30:30, w/w/w).
